# Neurotomy

**Published:** 1874-04

**Authors:** S. Weir Mitchell


*THE PHILADELPHIA MEDICAL TIMES—December,* 1873:

NEUROTOMY.—*By S. Weir Mitchell, M.D.*—In 1867, Letievant, after dividing the median nerve in a man, satisfied himself that both feeling and motion remained, where, according to the ordinary notions, none should have been found. A careful study, however, showed that each muscle fed by the cut nerve was really palsied, and that the motions seen were due to muscles supplied with nerve-force by the ulnar and radial. As to this there can be no doubt; and my own study of a recent case has on this point satisfied me thoroughly. The seeming presence of feeling was more of a puzzle. It was found that feeling was vastly lessened in the median area, but not lost, except in a very limited space; now, this state of things existed within a few hours after the section, when not even the wildest believer in regeneration of nerve could dream it had already taken place. The explanation in this especial case lies in the existence of branches from the ulnar which enter the median nerve below the point of section, and also in the intimate plexus it forms with other nerves at the fingers-ends. The anatomists are, in part, responsible for the clinical difficulty. Take, for instance, Flower's Atlas, or most of the anatomies, and you will see that the median innervates this arena, and the ulnar that, and so on, while in truth this whole surface anatomy is a fiction and has to be studied anew by closer dissections and by utilizing such nerve-sections as are made in man. In fact, I believe that it will be found that the regions of skin made sensitive by but one nerve-branch are very limited, and that throughout the whole surface, and not merely at the extremities, division of several nerve-stems will be needed to extinguish feeling of all forms in any one part of the skin. Strange, then, as it may seem, there is yet room for a careful monograph on the anastomoses of the main nerves.

I was amazed of late to see how difficult it is in man utterly to destroy feeling in the arm. In a notable case, Dr. Maury divided the whole brachial plexus in the neck. At first the posterior and inner cords were cut, and the patient was allowed to become free of the effects of ether. To my surprise, he still had touch-sense in the palm and dorsal surface of the hand, forearm, and arm, and on the inner face of the arm. The inner face of the forearm I think I did not examine. As to the rest, I am positive that partial feeling remained until, the external cord being divided, the whole sensibility was lost save at the upper parts of the arm, where there was, and still is, some tactile sense, due, I presume, to filaments given off from the plexus above the point of section. I do not dwell on the case, as it will be more fully related at another time. It went far towards justifying the extreme opinion I have mentioned as that likely to become a future medical belief. I had certainly believed, with others, that in the brachial plexus the interchange of fibres was notable, but I had not supposed it to be so perfect.

